# Potential Therapeutic Applications of Pulmonary Surfactant Lipids in the Host Defence Against Respiratory Viral Infections

**DOI:** 10.3389/fimmu.2021.730022

**Published:** 2021-09-27

**Authors:** Jianjian Ji, Ling Sun, Zichen Luo, Ying Zhang, Wang Xianzheng, Yingzhao Liao, Xie Tong, Jinjun Shan

**Affiliations:** ^1^ Jiangsu Key Laboratory of Pediatric Respiratory Disease, Institute of Pediatrics, Nanjing University of Chinese Medicine, Nanjing, China; ^2^ Genome Center of University of California Davis, National Institutes of Health (NIH) West Coast Metabolomics Center, Davis, CA, United States; ^3^ Pediatrics of Traditional Chinese Medicine, Shenzhen Traditional Chinese Medicine Hospital, Shenzhen, China

**Keywords:** pulmonary surfactant lipids, therapeutic applications, respiratory viral infections, COVID-19, ARDS

## Abstract

Pulmonary surfactant is a complex and highly surface-active material. It covers the alveolar epithelium and consists of 90% lipids and 10% proteins. Pulmonary surfactant lipids together with pulmonary surfactant proteins facilitate breathing by reducing surface tension of the air-water interface within the lungs, thereby preventing alveolar collapse and the mechanical work required to breathe. Moreover, pulmonary surfactant lipids, such as phosphatidylglycerol and phosphatidylinositol, and pulmonary surfactant proteins, such as surfactant protein A and D, participate in the pulmonary host defense and modify immune responses. Emerging data have shown that pulmonary surfactant lipids modulate the inflammatory response and antiviral effects in some respiratory viral infections, and pulmonary surfactant lipids have shown promise for therapeutic applications in some respiratory viral infections. Here, we briefly review the composition, antiviral properties, and potential therapeutic applications of pulmonary surfactant lipids in respiratory viral infections.

## Introduction

Pulmonary surfactant is a complex and highly surface-active material that are found in the fluid lining of the alveolar surface of the lungs (1). It forms a mobile-liquid phase that covers the alveolar epithelium to facilitate breathing by reducing surface tension at the air-water interface within the lungs, thereby preventing alveolar collapse and reducing the mechanical work required to breathe (1, 2). Pulmonary surfactant is an important lipoprotein complexes of the lung lining, consisting of 90% lipids and 10% proteins by weight, and it is produced predominantly by alveolar type 2 (ATII) cells (2, 3). Together with pulmonary surfactant proteins, lipids provide the surface activity of surfactants (2, 3). Pulmonary surfactant proteins contain four proteins, including surfactant protein (SP)-A, SP-B, SP-C, and SP-D. SP-B and SP-C are small hydrophobic peptides, while SP-A and SP-D are large, soluble, hydrophilic proteins that have key overlapping and distinct roles in innate immunity and the immunological homeostasis of the lung (1).

In addition to lowering surface tension and preventing alveolar collapse at end-expiration, pulmonary surfactant functions as a modulator of immune responses (1). Previous studies have revealed that pulmonary surfactant, especially pulmonary surfactant proteins, plays an important role in the host defence against respiratory tract infection (1). Most previous studies focused on the anti-infectious roles of SP-A and SP-D. These proteins were found to protect the lung against multiple viral infections by directly neutralising viruses and modulating host antiviral immunity (1). SP-A and SP-D were found to bind several viruses, including influenza A virus, respiratory syncytial virus (RSV), and human immunodeficiency virus, enhancing their clearance from mucosal points of entry and modulating the host antiviral immune response (4). Many studies have investigated the antiviral properties of pulmonary surfactant proteins possess antiviral effects; however, few studies have focussed on the antiviral properties of pulmonary surfactant lipids. Emerging data have shown that some pulmonary surfactant lipids potentiate the host defence against respiratory viral infections (3, 5). Because the surface of lung is permanently exposed to the virus and pro-inflammatory factors directly in the respiratory viral infections, it is particularly important to explore the host defence against viruses of pulmonary surfactant lipids. Herein, we briefly review the antiviral properties and relevant mechanisms of pulmonary surfactant lipids in respiratory viral infections and discuss their possible therapeutic applications.

## Pulmonary Surfactant Lipid Constituents and Functions

**Figure 1:**
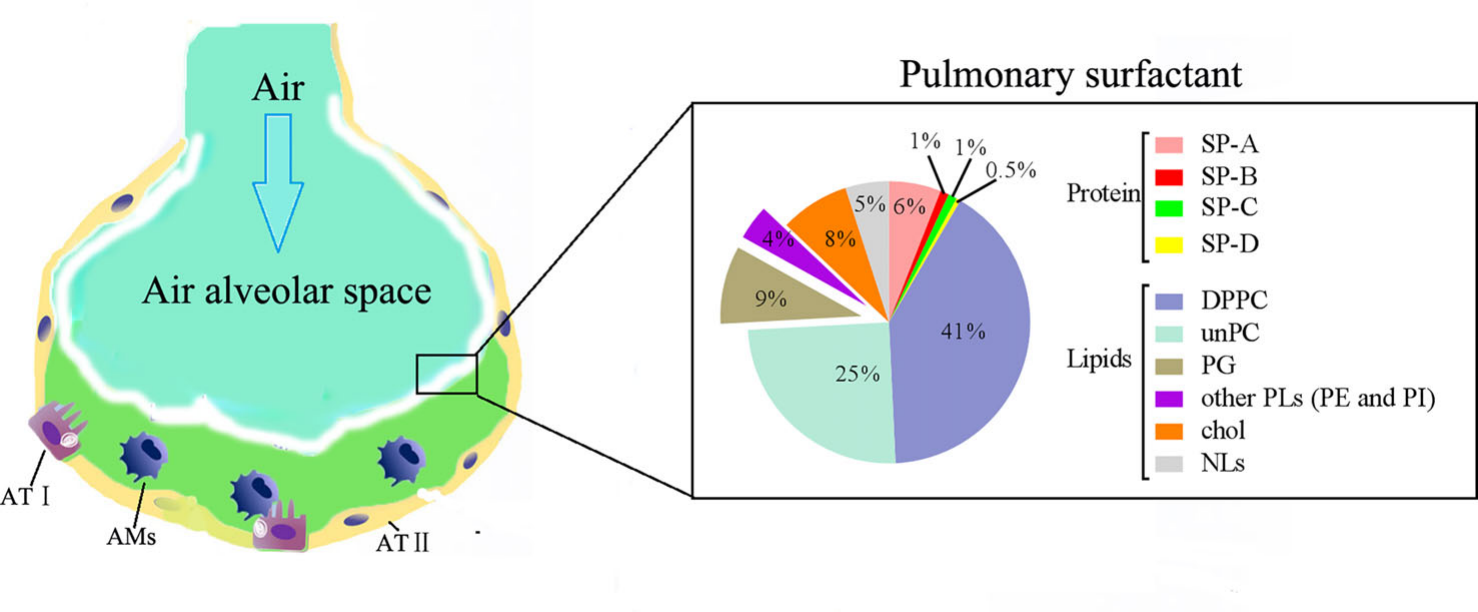
Pulmonary surfactant lipid constituents. Pulmonary surfactant components are important lipoprotein complexes of the lung lining, consisting of 90% lipids and 10% proteins by weight. Pulmonary surfactant proteins contain four proteins, including surfactant protein (SP)-A, SP-B, SP-C, and SP-D. In pulmonary surfactant lipids, the most abundant constituents are glycerophospholipids. Surfactant phospholipids (PLs) account for 80–85% of pulmonary surfactant lipids; surfactant PLs are a mixture of lipids, which include phosphatidylcholine (PC, accounting for about 80%), phosphatidylglycerol (PG, accounting for about 7–15%), and small quantities (accounting for approximately 5% each) of phosphatidylinositol (PI), phosphatidylethanolamine (PE), and phosphatidylserine (PS). The most prevalent PL in pulmonary surfactant is PC, and approximately 40% of surfactant PC is saturated dipalmitoyl-PC (DPPC).

In pulmonary surfactant lipids, the most abundant constituents are glycerophospholipids (2, 3). Surfactant phospholipids (PLs) account for 80–85% of pulmonary surfactant lipids, including phosphatidylcholine (PC, accounting for about 80%); phosphatidylglycerol (PG, accounting for about 7–15%); and small quantities (accounting for approximately 5% each) of phosphatidylinositol (PI), phosphatidylethanolamine (PE), and phosphatidylserine (PS) (**Figure 1**). The most prevalent PLs in pulmonary surfactant lipids is PC, and approximately 40% of pulmonary surfactant PC is saturated dipalmitoyl-PC (DPPC) (i.e., PC with two palmitic acid groups) (**Figure 1**). The tight intermolecular packing of DPPC, especially at end-expiration, is thought to be largely responsible for the surface tension-reducing activity of surfactants that guards against alveolar collapse (2, 3) as DPPC achieves very low surface tension upon compression (6). The remaining PC molecular species mainly include unsaturated lipids, such as 1-palmitoyl-2-oleoyl-sn-glycero-3-phosphocholine (POPC) (2, 3). The POPC in the surfactant film contributes to the membrane fluidity at physiological temperature, and unsaturated PC (PC16:0/16:1) is related to surface dynamics and respiratory rate (7). These unsaturated PCs improve the adsorption and spreading properties of surfactant at the air-liquid interface (8). Other pulmonary surfactant PLs such as PE, is important in facilitating/promoting curvature in some non-bilayer surfactant forms that are critical intermediates throughout the transitions from bilayers to interfacial films and their interconversions during surfactants metabolism (9, 10); and PI can increase the rate of alveolar fluid clearance and stabilise the surfactant monolayer (7).

In addition to preventing alveolar collapse during respiratory activity, pulmonary surfactant lipids can also modulate the inflammatory response to microbial components (3). PCs have anti-inflammatory properties that can alleviate tissue damage in multiple organs *via* the inhibition of multiple proinflammatory mediators. DPPC inhibits lipopolysaccharide (LPS)-induced cytokine production by airway epithelial cells and monocytes, and DPPC supplementation in mice attenuates lung inflammation. However, PONPC [1-palmitoyl-2-(9-oxononanayl)-PC], another component of PC, can increase the production of nitric oxide and cytokines in macrophages *via* the upregulation of *TLR4* and *Myd88* gene expression (11). PI and PG can inhibit macrophage proinflammatory cytokine responses to LPS. PG can also reduce inflammatory mediator production by blocking the toll-like receptor 2 (TLR2) pathway, thus repressing lung inflammation. Moreover, PG can inhibit the single-stranded RNA-activated TLR7/8 pathway and reduce pre-inflammation cytokine secretion. In a study, PG supplementation preserved lung function and prevented alveolar epithelial injury in a neonatal pig triple-injury model of acute respiratory distress syndrome (ARDS) (12). PLs competitively inhibit the binding of LPS to LPS-binding protein and CD14, which then inhibits the LPS–LPS-binding protein –TLR4 signalling pathway and attenuates inflammation (3).

## Alteration in Pulmonary Surfactant Lipids in Respiratory Viral Infections

Pulmonary surfactant lipids constitute the frontline of defence against inhaled pathogens (3). Respiratory viral infections, such as those caused by influenza virus and RSV, which are the most common respiratory viruses, can induce the dysfunction of pulmonary surfactant lipids metabolism (13). ATII cells are responsible for the synthesis, secretion and recycling of pulmonary surfactant (14), and they are the primary site of influenza virus replication in the distal lung (15). Influenza infection significantly alters ATII cells surfactant lipid metabolism, and this was reported to result in surfactant dysfunction and ARDS in influenza-infected mice (15). The levels of several major pulmonary surfactant PLs (PCs, PGs, and PEs) in ATII cells from influenza-infected mice were significantly decreased compared with that in mock-infected animals; however, the levels of several minor pulmonary surfactant lipids (PSs, PIs, and sphingomyelin), cholesterol, and diacylglycerol were increased in ATII cells from influenza-infected mice (15). Moreover, cytidine 5’-diphosphocholine and 5’-diphosphoethanolamine (liponucleotide precursors for PCs and PEs synthesis, respectively, in ATII cells) were also decreased (15). Furthermore, alterations in PLs in ATII cells were reflected in the composition of surfactant lipids in bronchial alveolar lavage fluid, which showed reduced amounts of PCs and PGs but increased amounts of sphingomyelin and cholesterol (15).

A study on lung tissue sample obtained from RSV-infected mice demonstrated alteration in 86 surfactant lipids, compared with that in control mice (16). Levels of PI, lyso-PI and plasmalogen lipids, including plasmenyl-PC and plasmenyl-PE were significantly elevated in the lungs of RSV-infected mice (16). The levels of palmitoylated PGs such as PG (16:0_22:5), PG (16:0_22:6), and PG (16:0_18:1) were decreased, but the levels of stearoylated PG lipids, such as PG (18:2_20:4), PG (18:2_18:2), and PG (18:1_20:4), were increased in the lung tissues of RSV-infected mice (14).

Although there is no convincing evidence that pulmonary surfactant lipids are dysfunctional in those with severe acute respiratory syndrome coronavirus 2 (SARS-CoV-2) infection, indirect evidence indicates that SARS-CoV-2 infection may induce alterations in the composition of pulmonary surfactant lipids in three ways. Firstly, SARS-CoV-2 infects ATII cells by binding angiotensin-converting enzyme 2 (17), and the infected cells provide an environment for SARS-CoV-2 replication. Colonisation of these cells by SARS-CoV-2 may interfere with the synthesis of pulmonary surfactant components. Secondly, SARS-CoV-2 infection may influence the recycling and catabolism of the used/spent/altered pulmonary surfactant in ATII cells and alveolar macrophages. Thirdly, inflammation can result in the compositional alterations of lipids (18). Therefore, the inflammations in the lung caused by SARS-CoV-2 infections may alter composition of pulmonary surfactant lipids. A previous study has shown that SARS-CoV-2 infections result in the decrease of pulmonary surfactant proteins (19), indicating that pulmonary surfactant lipid content may also be influenced by SARS-CoV-2. A recent study showed that the lipid metabolism in the plasma was altered in patients with coronavirus disease 2019 (COVID-19). The levels of PCs in plasma gradually reduced over time, while the levels of PEs and PSs in the plasma gradually increased over time in those with COVID-19 fatalities (20). Although the direct relationship between pulmonary surfactant and plasma lipids has not been studied, the composition of pulmonary surfactant lipid composition may be similarly altered in COVID-19 patients, and related research should emerge soon. Together, current evidence suggests that pulmonary surfactant lipid composition may undergo alterations following respiratory viral infection.

Altered pulmonary surfactant lipid composition not only influences surface tension-related properties but also impacts the progress of inflammation following viral infections. Importantly, recent studies have shown that supplementation with several pulmonary surfactant lipids, such as PGs (mainly POPG [1-palmitoyl-2-oleoyl-sn-glycero-3-phospho-(1′-rac-glycerol)] and PIs *via* intranasal inoculation can prevent some respiratory viral infections, and this may provide potential therapeutic applications for respiratory viral infections. This suggests potential therapeutic applications of pulmonary surfactant lipids for preventing or treating respiratory viral infections. We will briefly review these topics in the followings.

## Antiviral Effect of Pulmonary Surfactant Lipids in RSV Infection

RSV is a negative-sense, single-stranded RNA virus of the *Paramyxoviridae* family, and that is a leading cause of acute respiratory tract infections in early childhood (21). As mentioned earlier, the levels of some PGs in the lungs are decreased after RSV infection (16). Some studies have shown that POPG and PIs possess potent antiviral effects, and POPG supplementation can prevent RSV infection (5, 22–24). POPG can bind RSV with high affinity and inhibit virus attachment to cells; it then blocks viral plaque formation and markedly suppresses virus replication (22, 24). POPG can also attenuate inflammatory responses induced by RSV through direct interactions with the TLR4-interacting proteins, CD14 and MD-2 (5). Intranasal POPG supplementation significantly prevented virus infection and inflammation in the lungs of RSV-infected mice (22, 24). In addition, PI also markedly prevented RSV infection *in vivo* and *in vitro* (5, 23). The presence of PI during RSV challenges *in vitro* prevented virus attachment to epithelial cells by binding RSV with high affinity, blocking the spread of RSV from infected to uninfected cells and suppressing RSV replication (23). In another study, intranasal inoculation with PI reduced the viral load in lungs, eliminated the influx of inflammatory cells, and reduced lung tissue histopathology in RSV-infected mice (23). Collectively, these findings demonstrate that POPG and PI are effective for the prevention and treatment of RSV infections. Other studies indicate that the antiviral ability of POPG may be greater than that of PI, although the latter may confer longer-lasting protection against RSV infection (5).

Regarding the underlying mechanism(s), the antiviral effects of PI and POPG are achieved by their binding to RSV to block virus attachment to epithelial cells. However, it is unclear why PI and POPG have such a high affinity for RSV and how they bind to RSV. Moreover, it has not been determined whether the high affinity of PG and POPG is specific for RSV, or if this phenomenon applies to other viruses. It is also unknown if the antiviral mechanisms of PG and PI are the same. We believe these mechanisms should to be further explored as this information may be important for developing an effective strategy for controlling RSV infection.

## Antiviral Effects of Pulmonary Surfactant Lipids in Influenza Virus Infection

Influenza virus is one of the most common viruses globally, causing global health problems and life-threatening infections and resulting in an estimated 500,000 deaths each year (25). As mentioned above, the levels of some PCs and PGs were decreased in the lungs after influenza infection (15). Previous studies have shown that PG supplementation can suppress influenza virus infections (5, 26, 27). POPG can inhibit influenza A virus attachment to the plasma membrane and block subsequent replication *in vitro* (26). Another study showed that POPG can bind to two strains of influenza virus, H1N1-PR8-influenza and H3N2-influenza, with high affinity and block influenza virus replications (26, 27). Some studies revealed that the intranasal inoculation of POPG in H1N1-PR8-influenza-infected mice markedly reduced viral titres and suppressed inflammatory cell infiltrates in the lungs (5, 26, 27). PI can also bind to H1N1-influenza with high affinity and disrupt viral spread from infected to non-infected cells in tissue culture, reducing H1N1 propagation (5, 27). PI administration also significantly reduced lung inflammation and viral burden in infected mice (5, 27). These studies suggest that PI and PG can prevent influenza infection by binding to the influenza virus. The above studies also indicate that PI and PG are effective for preventing RSV infection. However, it is undetermined if the antiviral capabilities of PI and PG are against for most viruses or pertain only influenza and RSV.

## Antiviral Effects of Pulmonary Surfactant Lipids in Other Viral Infections

Previous studies showed that other pulmonary surfactant PLs, such as PC and PS, can control infection by reprogramming macrophages *via* negatively charged membrane (28). 1-stearoyl-2-arachidonoyl-PI (SAPI), which is the most abundant PI, can defend against dengue virus infection (29). DPPC can promote adenoviral entry into epithelial cells by binding the virus and serving as a vehicle for receptor-independent penetration into the cell (29). Exogenous PS also promotes cell entry by enveloped viruses, potentially by promoting fusion (30). Interestingly, PS in the poxvirus envelope promotes viral infectivity (31), possibly through apoptotic cell mimicry (32). Plasmalogen pre-conditioning may be potentially used as anti-viral therapeutic and prophylaxis strategy to treat SARS-CoV-2, influenza, human cytomegalovirus (HCMV) and West Nile Virus (WNV) infections (33). The potential anti-viral mechanism of plasmalogen may include influencing viral entry host cells *via* non-receptor microdomain mediated endocytosis pathways; modulating lipid-modulated host innate immune response and virus-induced host membrane rearrangements, especially cubic membrane (CM) formation (33).

As suggested above, not all lipids are protective against viral infection. A previous study showed that PE was required for the replication of a (+)RNA virus (34), and RNA virus replication depended on PE enrichment at replication sites in subcellular membranes (35). The PE receptor CD300a can bind dengue virus and enhance infection (36). Previous studies have shown that cholesterol play an important role in viral entry into host cells and cholesterol-lowering therapies can reduce viral infectivity (37). These studies suggest that not all lipids are protective in respiratory viral infection.

## Potential Therapeutic Applications of Pulmonary Surfactant Lipids in Respiratory Viral Infections

After respiratory viral infection, viruses can interfere with the synthesis and secretion of pulmonary surfactant; this can cause an increase in surface tension, leading to alveolar collapse and ARDS (38, 39). ARDS is characterised by lung inflammation and pulmonary oedema, which reduces gas exchange and leads to hypoxaemia and dyspnoea, with patients often requiring mechanical ventilation to provide sufficient oxygenation (38, 39). Pulmonary surfactant lipids can lower surface tension at the air-liquid interface, thus preventing alveolar collapse at end-expiration (1, 3). As such, supplementation with pulmonary surfactant lipids can effectively alleviate respiratory distress because of the lack of surfactant lipids in the lung. In fact, PG-containing surfactants have already been approved by the Food and Drug Administration for the treatment of neonatal respiratory distress syndrome (40). A previous study also showed that supplementation composed of surfactant with additional PG (to a molar percentage of 6%) preserved lung function and prevented alveolar epithelial injury in a neonatal pig triple-injury model of ARDS (12). Current studies have demonstrated that multiple respiratory viruses, such as RSV, SARS, and SARS-CoV-2, usually cause ARDS (38). Thus, pulmonary surfactant lipids supplementation does not only restore the decreased pulmonary surfactant lipids caused by viral infection, but it also reduces surface tension to decrease the work of breathing and increase oxygen supply.

Respiratory viral infections are accompanied by an aggressive proinflammatory cytokine response that is directly related to the severity of the disease (41). Thus, the inflammation modulatory function of pulmonary surfactant may be important for treating virus infection. Pulmonary surfactant lipids have been shown to modulate the inflammatory response to microbial components, such as LPS and single-stranded RNA, also known as pathogen-associated molecular patterns (PAMPs). Thus, supplementation with pulmonary surfactant lipids can effectively alleviate virus infection-induced inflammatory responses. Recently, several pulmonary surfactant lipids have been reported to have anti-inflammatory effects, among which PGs have been reported to play an anti-inflammatory role in many inflammatory processes (3, 5, 39). PGs were shown to inhibit the expression of interleukin (IL)-1α, IL-1β, IL-6, and/or TNFα, as well as IL-8 and interferon-γ in response to TLR activation (39), which then decreased inflammation in the lungs. Therefore, pulmonary surfactant lipids supplementation may reduce virus-induced inflammation.

**Figure 2:**
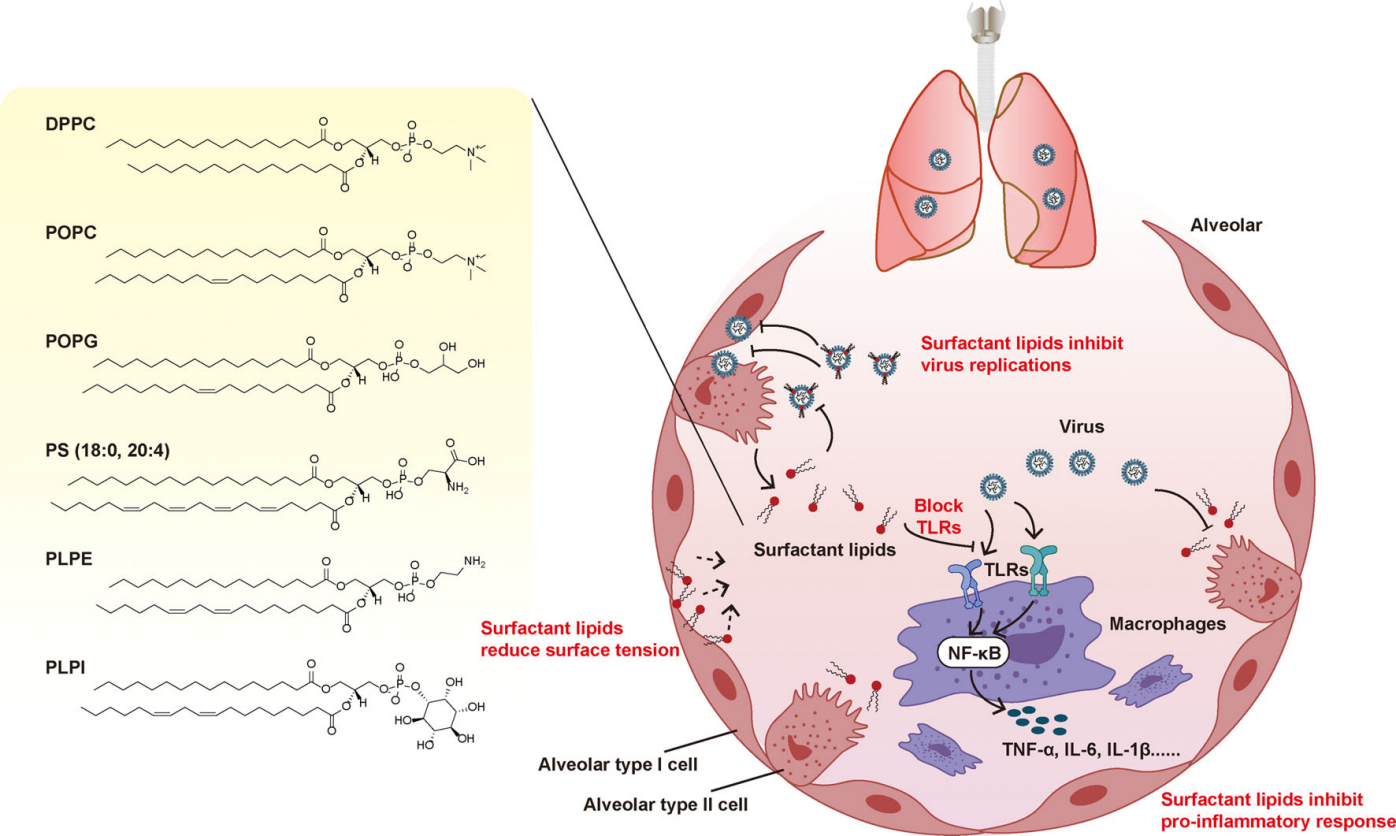
Potential mechanism of PLs in therapeutic applications in respiratory viral infections. Respiratory viral infections can induce the dysfunction of pulmonary surfactant lipids. Supplement the pulmonary surfactant lipids may have therapeutic effects as follows: it may (1) supplement the decreased pulmonary surfactant lipids; (2) reduce surface tension and prevent alveolar collapse during respiratory activity; (3) inhibit the proinflammatory response and alleviate tissue damage in lungs; and (4) inhibit virus replications and limit virus infection.

Importantly, recent studies have shown that POPG and PI supplementation can combat RSV and influenza infection by blocking viral replication (5). Although, it is unclear whether pulmonary surfactant lipids can defend against other respiratory viral infections, this suggests it is worthwhile to explore the antiviral effect of pulmonary surfactant lipids. Another application of pulmonary surfactant lipids is using them as a vehicle for antiviral drugs administrated by the pulmonary route. Use surfactant lipids as a vehicle can offer compatibility for delivering antiviral drugs, vaccines and other therapeutic molecules, which enhances targeted delivering and also has capability for immunomodulation (42). For example, DPPC liposomes can also be loaded with hydroxychloroquine to treat COVID‐19 disease trough inhalation (43). Collectively, the pulmonary administration of exogenous pulmonary surfactant lipids may have therapeutic effects as follows (**Figure 2**): the pulmonary surfactant lipids may (1) supplement the decreased pulmonary surfactant lipids; (2) reduce surface tension and prevent alveolar collapse during respiratory activity; (3) inhibit the proinflammatory response and alleviate tissue damage in lungs; and (4) inhibit virus replications and limit viral infection;(5) be as a vehicle for drugs administrated by the pulmonary route.

However, some pulmonary surfactant lipids such as PEs may facilitate RNA virus infection. Thus, not all pulmonary surfactant lipids can be used to treat viral infections. As pulmonary surfactant lipids contain many species and subclasses, further studies need to be performed to explore the potential functions of each pulmonary surfactant lipid in respiratory viral infections.

It is worth noting that, pulmonary surfactant proteins also possess anti-viral and anti-inflammatory properties, especially SP-A and SP-D (4). Therefore, the use of pulmonary surfactant lipids in combination with pulmonary surfactant proteins may be more effective in treatment of respiratory virus infection. In fact, current studies have used a combination of pulmonary surfactant lipids and pulmonary surfactant proteins to treat ARDS (44).

## Potential Therapeutic Applications of Pulmonary Surfactant Lipids in SARS-CoV-2 Infection

Alterations in pulmonary surfactant composition may occur in COVID-19 patients; thus, the administration of pulmonary surfactant lipids may be effective in COVID-19 patients. Several groups have undertaken studies to investigate the therapeutic value of exogenous pulmonary surfactant lipids in COVID-19 patients (39). A clinical trial of surfactants treatment on COVID-19 patients was ongoing (45). In this trail, a natural animal derived (bovine) lung surfactants, Bovactant (Alveofact^®^) was used and it consisted of a mixture of phospholipid (75% PCs, 13% PG, 3% PE, 1% PI and 1% sphingomyelin), 5% cholesterol, 1% surfactant proteins (SP-B and SP-C), very low levels of free fatty acid, lyso-phosphatidylcholine, water and 0.3% calcium (45). Whether pulmonary surfactant lipids possess antiviral effects against SARS-CoV-2 infection is still unknown, and we hope future studies will soon reflect on this subject.

COVID-19 is usually accompanied by ARDS, which may result in severe inflammation, multiorgan failure, and death (17). Because there are no specific antiviral treatments for SARS-CoV-2 infection, it is necessary to find alternative supportive treatments to prevent ARDS, severe inflammation, and pulmonary failure (39), which are the most common causes of COVID-19 mortality. The use of the pulmonary surfactant seems to be promising as an additional therapy for the treatment of ARDS and has been proposed by some researchers (39, 40). They concluded that pulmonary surfactant lipids supplementation could potentially reduce surface tension, inhibit the proinflammatory response, and improve ARDS in COVID-19, and we agree with their opinion based on the above discussion. However, some lipids, such as PE and cholesterol, could facilitate virus infection. As a result, it is important to clarify the alterations of pulmonary surfactant components in COVID-19 before conducting related trials.

## Conclusions

In summary, pulmonary surfactant lipids have multiple functions beyond simply reducing the surface tension and altering the mechanical properties of the lung. Notably, these additional functions include anti-inflammatory and antiviral roles in the lungs. As the lung epithelium is constantly exposed to the environment, pulmonary surfactant provides a crucial first line of defence against infection by enhancing the removal of pathogens, modulating the response of inflammatory cells, and optimising lung biophysical activity. Compared to the studies on the application of pulmonary surfactant proteins in viral infections, current studies on pulmonary surfactant lipids are still in early stages, and few in number. Therefore, further studies are required to explore the possibility of pulmonary surfactant lipids as a therapeutic approach or developmental drug therapy in respiratory viral infections. Taken together, this review can form the basis to guide future studies regarding research directions for the study of pulmonary surfactant lipids.

## Author Contributions

JJ and LS wrote the manuscript. ZL, WX, and YL assisted with the manuscript preparation. YZ, XT, and JS revised and polished the manuscript. All authors contributed to the article and approved the submitted version.

## Funding

This work was supported by the National Natural Science Foundation of China (No. 82004204 and 81774156); and sponsored by Qing Lan Project, and Natural Science Foundation of Nanjing University of Chinese Medicine (No. NZY82004204), The Open Projects of the Discipline of Chinese Medicine of Nanjing University of Chinese Medicine Supported by the Subject of Academic priority discipline of Jiangsu Higher Education Institutions (ZYX03KF053, ZYX03KF050), Natural Science Foundation of Jiangsu Province of China (Grant number BK20180825); Graduate Student Scientific Research Innovation Projects in Jiangsu Province (SJCX21-0696).

## Conflict of Interest

The authors declare that the research was conducted in the absence of any commercial or financial relationships that could be construed as a potential conflict of interest.

## Publisher’s Note

All claims expressed in this article are solely those of the authors and do not necessarily represent those of their affiliated organizations, or those of the publisher, the editors and the reviewers. Any product that may be evaluated in this article, or claim that may be made by its manufacturer, is not guaranteed or endorsed by the publisher.

## Abbreviations

ARDS, acute respiratory distress syndrome; ATII, alveolar type 2; COVID-19, coronavirus disease 2019; DPPC, dipalmitoyl- phosphatidylcholine; LPS, lipopolysaccharide; PC, phosphatidylcholine; PE, phosphatidylethanolamine; PG, phosphatidylglycerol; PI, phosphatidylinositol; PL, phospholipid; PONPC, 1-palmitoyl-2-(9-oxononanayl)-phosphatidylcholine; POPC, 1-palmitoyl-2-oleoyl-sn-glycero-3-phosphocholine; POPG, 1-palmitoyl-2-oleoyl-sn-glycero-3-phospho-(1’-rac-glycerol); PS, phosphatidylserine; RSV, respiratory syncytial virus; SAPI, 1-stearoyl-2-arachidonoyl-phosphatidylinositol; SARS-CoV-2, severe acute respiratory syndrome coronavirus 2; SP, surfactant protein; TLR, toll-like receptor; HCMV, human cytomegalovirus; WNV, West Nile Virus.
